# Supplementary material for: Saccharomyces boulardii (CNCM I-745) improves intestinal damage in sepsis by remodeling intestinal flora structure
Source: Front Cell Infect Microbiol. 2025 Jul 31;15:1602792. doi: 10.3389/fcimb.2025.1602792 (PMC12350329; doi:10.3389/fcimb.2025.1602792)
Supplement: Supplementary file 1 [file DataSheet1.zip › summary/summary/7_Differential_analysis/1_diff_abundance/6_Genus/Group.DW4_vs_BW4/Group.DW4_vs_BW4_diff_barplot.pdf]

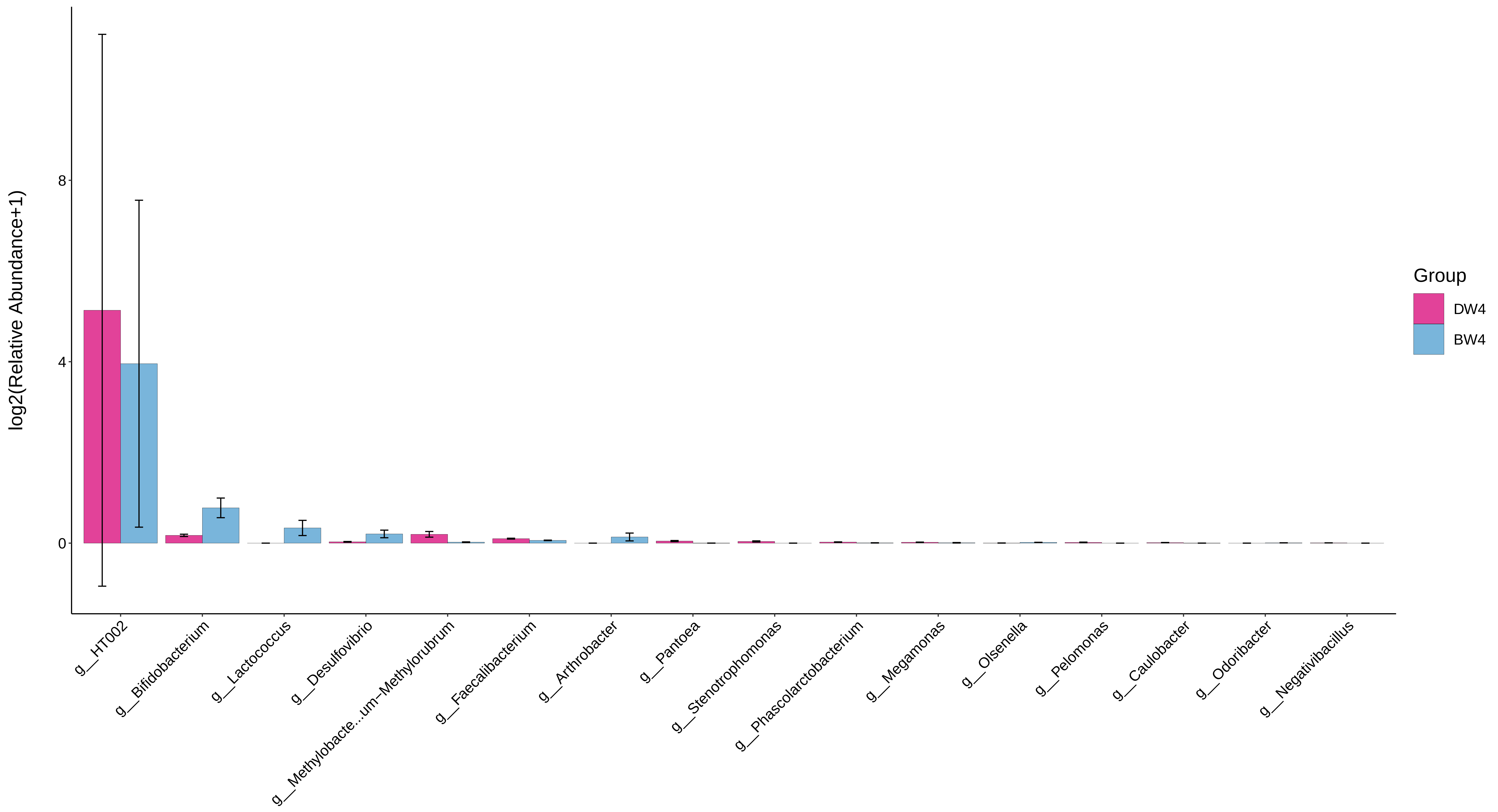
log2(Relative Abundance+1)
8
4
0
g__HT002
g__Bifidobacterium
g__Lactococcus
g__Desulfovibrio
g__Methylobacte...um−Methylorubrum
g__Faecalibacterium
g__Arthrobacter
g__Pantoea
g__Stenotrophomonas
g__Phascolarctobacterium
g__Megamonas
g__Olsenella
g__Pelomonas
g__Caulobacter
g__Odoribacter
g__Negativibacillus
Group
DW4
BW4
